# Alcohol Use and Co-Use of Other Substances Among Pregnant Females Aged 12–44 Years — United States, 2015–2018

**DOI:** 10.15585/mmwr.mm6931a1

**Published:** 2020-08-07

**Authors:** Lucinda J. England, Carolyne Bennett, Clark H. Denny, Margaret A. Honein, Suzanne M. Gilboa, Shin Y. Kim, Gery P. Guy, Emmy L. Tran, Charles E. Rose, Michele K. Bohm, Coleen A. Boyle

**Affiliations:** ^1^Division of Birth Defects and Infant Disorders, National Center on Birth Defects and Developmental Disabilities, CDC; ^2^Eagle Global Scientific, LLC, San Antonio, Texas; ^3^Division of Overdose Prevention, National Center for Injury Prevention and Control, CDC; ^4^Office of the Director, National Center on Birth Defects and Developmental Disabilities, CDC; ^5^Division of Population Health, National Center for Chronic Disease Prevention and Health Promotion, CDC.

Drinking alcohol during pregnancy can cause fetal alcohol spectrum disorders, including birth defects, behavioral disorders, and impaired cognitive development (*1*). Little is known about the co-use of other substances by females who drink during pregnancy. CDC used 2015–2018 data from the National Survey on Drug Use and Health (NSDUH) to estimate the overall and trimester-specific prevalence of self-reported drinking in the past 12 months, current drinking, and binge drinking, overall and by trimester, and the co-use of other substances among pregnant females aged 12–44 years. Past drinking (12 months) was reported by 64.7% of pregnant respondents. Current drinking (at least one drink in the past 30 days) was reported by 19.6% of respondents who were in their first trimester of pregnancy and 4.7% of respondents who were in their second or third trimester. Binge drinking (consuming four or more drinks on at least one occasion in the past 30 days) was reported by 10.5% of first trimester respondents and 1.4% of second or third trimester respondents. Overall, 38.2% of pregnant respondents who reported current drinking also reported current use of one or more other substances. The substances used most with alcohol were tobacco and marijuana. Self-reported drinking prevalence was substantially lower among second or third trimester respondents than among first trimester respondents. The American College of Obstetricians and Gynecologists (ACOG) recommends alcohol use and substance use disorders screening for all females seeking obstetric-gynecologic care and counseling patients that there is no known safe level of alcohol use during pregnancy (*2*).

NSDUH is a nationwide survey that uses multistage and area probability sampling to provide information on tobacco, alcohol, and drug use, and on mental health and other health-related issues, among U.S. civilian, noninstitutionalized persons aged ≥12 years. Surveys are conducted in respondents’ homes and use computer-assisted interviewing methods. Female respondents report whether they are currently pregnant and the trimester of pregnancy at the time of the interview. Weighted response rates for 2015–2018 ranged from 66.6% to 69.3%.*

This report focuses on past 12 months drinking, current drinking and binge drinking among pregnant respondents. Drinking alcohol during pregnancy and binge drinking in any population are two measures of excessive drinking.^†^ In addition, this report provides estimates of the prevalence of co-use of other substances among respondents who drank alcohol. Respondents who reported ever having an alcoholic beverage were asked how long it had been since they last drank an alcoholic beverage.

This report also examined past 12 months and past 30 days use of other substances, including tobacco (i.e., cigarettes, cigars, smokeless tobacco, and pipes), marijuana, opioids (prescription pain reliever misuse and heroin use), and “other substances,” which included cocaine, hallucinogens, inhalants, methamphetamines, and the misuse^§^ of sedatives, stimulants, and tranquilizers. Other substances were grouped as one category because of the small number of pregnant females who reported using them.

Data were weighted to adjust for nonresponse and to generate nationally representative estimates. Prevalence estimates and 95% confidence intervals (CIs) for past 12 months drinking, current drinking, and binge drinking were calculated overall and by sociodemographic and pregnancy characteristics (age, race/ethnicity, income, marital status, education, employment status, insurance status, county urban/rural status, and trimester of pregnancy). Prevalence estimates and 95% CIs for past 12 months and current drinking alone and with co-occurring substance use among pregnant respondents also were calculated. Analyses were conducted using SAS (version 9.4; SAS Institute) with SUDAAN (version 11.0; RTI International) to account for the complex sampling method used in NSDUH. This activity was reviewed by CDC and conducted consistent with CDC policies and procedures.^¶^

Among 99,618 female respondents aged 12–44 years, 3,006 (3%) reported a current pregnancy. Among pregnant respondents, past 12 months drinking, current drinking, and binge drinking prevalence estimates were 64.7%, 9.8%, and 4.5%, respectively (Table 1). Past 12 months drinking was reported by 76.1% of first trimester respondents and 59.8% of second or third trimester respondents; current drinking by 19.6% of first trimester respondents and 4.7% of second or third trimester respondents; and binge drinking by 10.5% of first trimester respondents and 1.4% of second or third trimester respondents (p<0.001 for all comparisons) (Table 1; Figure).

**TABLE 1:** Weighted prevalence of past 12 months and past 30 days drinking and past 30 days binge drinking in 3,006 pregnant females aged 12–44 years, by selected characteristics — National Survey on Drug Use and Health, United States, 2015–2018

Characteristic	% (95% CI)		
	Past 12 months drinking*	Past 30 days drinking*	Past 30 days binge drinking*
**Overall**	64.7 (62.1–67.3)	9.8 (8.5–11.1)	4.5 (3.7–5.4)
**Age group (yrs)**			
<18	39.0 (27.3–52.1)	—^†^	—^†^
18–25	61.2 (58.0–64.3)	9.9 (7.9–12.2)	6.0 (4.5–8.1)
26–34	68.2 (64.2–72.0)	9.4 (7.6–11.5)	3.9 (2.9–5.3)
≥35	63.1 (55.5–70.1)	11.1 (7.3–16.6)**^§^**	—^†^
**Race/Ethnicity**			
White, non-Hispanic	74.9 (71.4–78.0)	9.9 (8.2–11.8)	4.0 (3.0–5.3)
Black, non-Hispanic	56.7 (51.0–62.3)	13.7 (9.8–18.9)	7.0 (4.6–10.9)**^§^**
Hispanic	48.0 (42.2–53.7)	7.0 (4.5–10.7)**^§^**	—^†^
Other	52.9 (44.0–61.7)	8.4 (4.8–14.4)	—^†^
**Income** ^§^			
<$20,000	50.5 (45.2–55.8)	9.7 (7.3–12.8)	6.3 (4.7–8.3)
$20,000–$74,999	61.6 (57.9–65.2)	8.7 (7.0–10.8)	3.9 (2.8–5.4)
≥$75,000	78.3 (74.0–82.1)	11.4 (9.2–14.0)	4.3 (3.0–6.0)
**Marital status** ^¶^			
Married	66.2 (62.2–70.0)	9.0 (7.3–11.0)	3.1 (2.2–4.3)
Not married	63.6 (59.9–67.1)	11.0 (9.1–13.3)	6.5 (5.0–8.4)
**Education** ^¶^			
≤High school	49.2 (45.3–53.0)	8.9 (6.9–11.4)	5.3 (3.8–7.6)
>High school	73.3 (70.0–76.3)	10.3 (8.8–12.0)	4.0 (3.1–5.2)
**Employment** ^¶^			
Full time	76.3 (73.1–79.1)	11.6 (9.7–13.9)	4.6 (3.4–6.2)
Part time	62.5 (57.2–67.5)	8.7 (5.8–12.8)	3.4 (1.9–6.2)**^§^**
Unemployed/Other**	53.5 (49.3–57.7)	8.3 (6.4–10.6)	4.8 (3.4–6.6)
**Insurance**			
Medicaid	54.9 (51.3–58.5)	7.6 (6.0–9.5)	3.8 (2.8–5.0)
Private	73.9 (70.2–77.3)	10.6 (8.6–13.0)	4.0 (3.0–5.5)
Uninsured/Other**^††^**	53.2 (46.2–60.1)	13.5 (9.4–18.9)	9.5 (5.7–15.4)**^§^**
**Urban/Rural** ^§§^			
Metropolitan	65.7 (61.6–69.5)	10.3 (8.4–12.7)	3.8 (2.7–5.4)
Micropolitan	64.5 (60.6–68.2)	9.3 (7.1–12.0)	4.9 (3.7–6.6)
Rural	61.0 (55.8–66.0)	8.3 (5.9–11.7)	6.3 (3.9–9.8)**^§^**
**Trimester** ^¶¶^			
First	76.1 (72.7–79.3)	19.6 (16.8–22.7)	10.5 (8.5–13.0)
Second or third	59.8 (56.5–63.1)	4.7 (3.5–6.4)	1.4 (0.9–2.1)

**Abbreviation:** CI = confidence interval.

* Past 12 months use regardless of whether there was also past 30 days use; past 30 days drinking regardless of whether there was also past 30 days binge drinking; binge drinking = consuming four or more drinks on at least one occasion in the past 30 days.

^† ^Estimates are not presented because the relative standard error was >30%.

**^§ ^**Estimate might be unstable because the relative standard error is 20%–30%.

^¶ ^The age group <18 years was omitted for income, marital status, education, and employment

** Other = those not in the labor force.

**^†† ^**Other insurance not otherwise specified.

^§§^https://www.ers.usda.gov/data-products/rural-urban-continuum-codes.

^¶¶ ^Overall, 1.3% of females reported an unknown trimester of pregnancy and were not included in the table.

**FIGURE:**
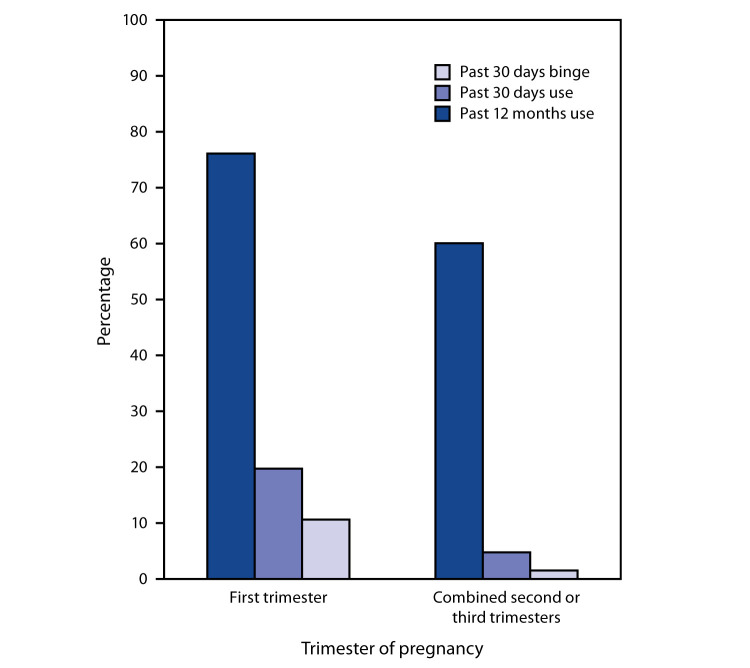
Weighted prevalence of past 12 months drinking, past 30 days drinking, and past 30 days binge drinking* among pregnant females^†^ aged 12–44 years (N = 3,006), by trimester — National Survey on Drug Use and Health, United States, 2015–2018 * For females, binge drinking = four or more drinks per occasion. ^†^ Overall, 1.3% of pregnant females reported an unknown trimester of pregnancy and were not included in the figure.

Among respondents who were pregnant and reported drinking in the past 12 months, 41.7% also reported using at least one other substance in the past 12 months. The most commonly reported substances were tobacco (30.3%), marijuana (21.9%), and opioids (7.0%) (Table 2). Among respondents who reported current drinking, 38.2% reported using at least one other substance, most commonly tobacco (28.1%) and marijuana (20.6%) (Table 2)

**TABLE 2:** Weighted prevalence of substance use patterns (past 12 months and past 30 days) in pregnant females aged 12–44 years (N = 3,006*) who drank alcohol in the past 12 months (n = 1,851*) or the past 30 days (n = 282*) — National Survey on Drug Use and Health, United States, 2015–2018

Substance use pattern	% (95% CI)	
	Past 12 months drinking^†^	Past 30 days (current) drinking
All pregnant females (N = 3,006*)		
**Any alcohol use**	64.7 (62.1–67.3)	9.8 (8.5–11.1)
**Alcohol use only**	37.7 (35.7–39.7)	6.0 (5.0–7.2)
**Alcohol and ≥1 additional substance**	27.0 (25.1–29.0)	3.7 (2.9–4.7)
**Other substances used^§^**		
Tobacco^¶^	19.6 (18.0–21.3)	2.7 (2.1–3.6)
Marijuana	14.2 (12.3–16.3)	2.0 (1.4–2.8)
Opioids**	4.5 (3.5–5.8)	—^††^
Other^††^	6.2 (5.0–7.7)	—^††^
Pregnant females who drank in the past 12 months (n = 1,851*) or in the past 30 days (n = 282*)		
**Alcohol use only**	58.3 (56.0–60.6)	61.8 (53.9–69.2)
**Alcohol and ≥1 additional substance**	41.7 (39.4–44.0)	38.2 (30.8–46.1)
**Other substances used^§^**		
Tobacco^¶^	30.3 (28.0–32.8)	28.1 (21.7–35.6)
Marijuana	21.9 (19.0–25.0)	20.6 (14.5–28.3)
Opioids**	7.0 (5.5–8.9)	—^††^
Other^§§^	9.76 (7.8–11.8)	—^††^

**Abbreviation:** CI = confidence interval.

* Unweighted.

^†^ Past 12 months use, regardless of whether there was also drinking in the past 30 days (current drinking).

**^§ ^**Not mutually exclusive.

^¶ ^Includes cigarettes, cigars, or smokeless tobacco.

** Includes prescription pain reliever misuse and heroin use.

^†† ^Estimates are not presented because the relative standard error was >30%.

**^§§^** Includes use of cocaine, hallucinogens, inhalants, methamphetamines, and the misuse of sedatives, stimulants, and tranquilizers.

Overall, 19.6% of respondents who were pregnant reported past 12 months drinking and tobacco use, 14.2% reported past 12 months drinking and marijuana use, 4.5% reported past 12 months drinking and opioid use, 2.7% reported current drinking and tobacco use, and 2.0% reported current drinking and marijuana use.

## Discussion

During 2015–2018, approximately half of all pregnant respondents who reported current drinking (drinking in the past 30 days) (9.8%) also reported binge drinking (4.5%). Among pregnant females who reported current drinking, 38.2% also reported current use of one or more other substances, including tobacco, marijuana, opioids, and other substances. The estimates of current drinking and binge drinking among pregnant females are consistent with recent analyses using data from the 2015–2017 Behavioral Risk Factor Surveillance System, which reported current drinking and binge drinking estimates of 11.5% and 3.9%, respectively, among pregnant respondents aged 18–44 years (*3*). The current analysis adds to previous findings by including trimester-specific estimates showing higher self-reported drinking in first trimester respondents, suggesting that some respondents who drank before pregnancy might have quit by mid-to-late pregnancy, and by providing estimates indicating that co-use of other substances is common.

Few population-based reports consider co-use of other substances among pregnant females who drink alcohol. In this report, current drinking overall and in combination with one or more other substances were substantially lower than past 12 months drinking, suggesting that females decrease their use after they know they are pregnant. Alcohol exposure during pregnancy can adversely affect fetal development, resulting in behavioral disorders, impaired intellectual development, and birth defects (*1*). It also has been associated with miscarriage and stillbirth (*4*). Although supporting data are sparse, alcohol exposure combined with exposure to other substances could worsen pregnancy outcomes. Prenatal exposure to substances included in this analysis has been associated with adverse health outcomes, including preterm birth, sudden infant death syndrome, and preterm-related death (exposure to tobacco) (*5*); low birth weight (tobacco, marijuana) (*5**,**6*); and altered fetal brain development (tobacco, marijuana) (*5*–*8*). A review of prenatal substance exposure and neuroimaging suggests that in utero exposure to substances other than alcohol, including marijuana, nicotine, cocaine, methamphetamine, opioids, or combinations of substances, is associated with long-term effects on cognition and with altered brain connectivity and white matter deficits (*9*).

The findings in this report are subject to at least four limitations. First, data are self-reported and therefore subject to social desirability bias; respondents might underreport substance use because of social stigma and legal implications. Second, because NSDUH only ascertains past 12 months and past 30 days substance use in a cross-sectional sample, patterns across individual pregnancies are unknown. Estimates of any substance use during the length of an entire pregnancy would likely be higher than estimates of past 30 days use. Third, limited sample size necessitated the suppression of some prevalence estimates. Finally, some pregnancies might not have been recognized at the time of the interview, resulting in misclassification by pregnancy status. Alcohol use and other substance use presumably are lower in recognized than in unrecognized pregnancies, resulting in underestimation of exposure levels.

The U.S. Preventive Services Task Force recommends alcohol screening and brief behavioral counseling in primary care settings for all adults aged ≥18 years (*10*). ACOG recommends alcohol use screening for all females seeking obstetric-gynecologic care and counseling patients that there is no known safe level of alcohol use during pregnancy (*2*). ACOG also recommends routine universal screening for substance use disorders with validated screening tools or through conversations with patients. Although ACOG does not have recommendations specific to polysubstance use, the findings of this report indicate that a substantial percentage of females who use alcohol during early pregnancy also use one or more other substances, especially tobacco or marijuana. Females could benefit from screening and interventions in pregnancy to reduce alcohol and polysubstance use and from referral for those in need of treatment. Successful reduction in substance exposures during pregnancy could improve the health of mothers and their children.

**Summary**

**What is already known about this topic?**

Drinking alcohol during pregnancy can cause miscarriage, stillbirth, and fetal alcohol spectrum disorders; however, approximately one in nine pregnant females report current drinking. Little is known about the co-use of other substances by females who drink during pregnancy.

**What is added by this report?**

Pregnant respondents in the first trimester reported higher current alcohol use than respondents in the second or third trimester. Among first trimester respondents, 19.6% reported current alcohol use and 10.5% reported binge drinking; among second or third trimester respondents, current drinking and binge drinking were reported by 4.7% and 1.4%, respectively. Approximately 40% of pregnant females reporting current drinking also reported current use of other substances.

**What are the implications for public health?**

Co-use of other substances is common among females who drink alcohol during pregnancy. Screening and interventions for alcohol and other substances in pregnancy could improve the health of mothers and their children.

## Footnotes

* https://www.samhsa.gov/data/sites/default/files/NSDUH-MethodSummDefsHTML-2015/NSDUH-MethodSummDefsHTML-2015/NSDUH-MethodSummDefs-2015.htm.

† Excessive drinking by females includes binge drinking (four or more drinks per occasion), heavy drinking (more than one drink per day on average), any drinking by pregnant females, and drinking by females aged <21 years. https://health.gov/dietaryguidelines/2015/.

§ Misuse includes 1) use without a prescription of the respondent's own medication; 2) use in greater amounts, more often, or longer than the respondent was told to take them; or 3) use in any other way a doctor did not direct the respondent to use them.

¶ U.S. Department of Health and Human Services, Title 45 Code of Federal Regulations 46, Protection of Human Subjects.
