# *Erratum*: Vol. 69, No. 31

**DOI:** 10.15585/mmwr.mm6934a7

**Published:** 2020-08-28

In the report “Alcohol Use and Co-Use of Other Substances Among Pregnant Females Aged 12–44 Years — United States, 2015–2018,” on p. 1011, errors occurred in the “Race/Ethnicity” section of Table 1. In the row for “Black, non-Hispanic,” the numbers under “Past 30 days binge drinking*” should have read **7.2 (4.6–10.9)^§^** and in the row for “Other,” the numbers under “Past 30 days drinking*” should have read **8.4 (4.8–14.4)^§^**.

